# Regulation of Class A β-Lactamase CzoA by CzoR and IscR in *Comamonas testosteroni* S44

**DOI:** 10.3389/fmicb.2017.02573

**Published:** 2017-12-22

**Authors:** Weiping Zhuang, Hongliang Liu, Jingxin Li, Lu Chen, Gejiao Wang

**Affiliations:** ^1^State Key Laboratory of Agricultural Microbiology, College of Life Science and Technology, Huazhong Agricultural University, Wuhan, China; ^2^Shandong Provincial Research Center for Bioinformatic Engineering and Technique, School of Life Sciences, Shandong University of Technology, Zibo, China

**Keywords:** *Comamonas testosteroni*, CzoR, IscR, cephalosporin resistance, Class A β-lactamase

## Abstract

A genomic analysis of *Comamonas testosteroni* S44 revealed a gene that encodes a LysR family transcriptional regulator (here named *czoR*, czo for cefazolin) located upstream of a putative class A β-lactamase encoding gene (here named *czoA*). A putative DNA-binding motif of the Fe–S cluster assembly regulator IscR was identified in the *czoR*–*czoA* intergenic region. Real-time RT-PCR and *lacZ* fusion expression assays indicated that transcription of *czoA* and *czoR* were induced by multiple β-lactams. CzoA expressed in *Escherichia coli* was shown to contribute to susceptibility to a wide range of β-lactams judged from minimum inhibitory concentrations. *In vitro* enzymatic assays showed that CzoA hydrolyzed seven β-lactams, including benzylpenicillin, ampicillin, cefalexin, cefazolin, cefuroxime, ceftriaxone, and cefepime. Deletion of either *iscR* or *czoR* increased susceptibility to cefalexin and cefazolin, while complemented strains restored their wild-type susceptibility levels. Electrophoretic mobility shift assays (EMSA) demonstrated that CzoR and IscR bind to different sites of the *czoR*–*czoA* intergenic region. Precise CzoR- and IscR-binding sites were confirmed via DNase I footprinting or short fragment EMSA. When cefalexin or cefazolin was added to cultures, *czoR* deletion completely inhibited *czoA* expression but did not affect *iscR* transcription, while *iscR* deletion decreased the expressions of both *czoR* and *czoA.* These results reveal that CzoR positively affects the expression of *czoA* with its own expression upregulated by IscR.

## Introduction

β-Lactam antibiotics are currently one of the most widely used antibacterial classes to treat infectious diseases, with cephalosporins (e.g., cefalexin, cefazolin, and cefradine) accounting for nearly half of all β-lactam antibiotic prescriptions (Bush and Bradford, 2016). Resistance of pathogenic bacteria to β-lactams has led to antibiotic treatment failures (Bush, 2013). Moreover, a number of environmental isolates have also shown a highly intrinsic or adaptive resistance to antibiotics. Production of β-lactamases is the primary mechanism of β-lactam resistance (Giwercman et al., 1992; Bush, 2013). Based on amino acid sequences, β-lactamases have been classified into four molecular classes. Classes A, C, and D β-lactamases hydrolyze their substrates through an active site serine, whereas Class B β-lactamases are metalloenzymes that require divalent zinc ions for their activities (Walsh et al., 2005).

A large number of β-lactamases have been reported, among which the Class A enzymes are the most abundant (Bush and Jacoby, 2010). The substrates of these class A β-lactamases are mostly penicillins, monobactams, early cephalosporins, and extended-spectrum cephalosporins, and these enzymes are inhibited by β-lactamase inhibitors, such as clavulanic acid, tazobactam, and sulbactam (Bush and Jacoby, 2010). Diverse class A β-lactamases have been studied for various genera, including GIL-1 from *Citrobacter gillenii*, AST-1 from *Nocardia asteroids*, PenA from *Burkholderia cepacia*, NmcA from *Enterobacter cloacae*, CumA from *Proteus vulgaris*, SmeA from *Serratia marcescens*, CdiA from *Citrobacter diversus*, Sed-1 from *Citrobacter sedlakii*, HugA from *Proteus penneri*, and BlaA from *Yersinia enterocolitica* (see review of Philippon et al., 2016).

Underlying regulatory mechanisms of β-lactamases have been largely focused on the class C β-lactamase AmpC, which is regulated by the LysR family transcriptional AmpRs regulator of *Citrobacter freundii* and *E. cloacae* (Lindquist et al., 1989; Guérin et al., 2015). In the presence of β-lactams, an excessive breakdown of murein leads to the accumulation of AmpD-unprocessed muramyl peptides presumably, but not β-lactam itself, binding AmpR, which induces a conformational change in AmpR to promote expression of *ampC* (Jones and Bennett, 1995; Caille et al., 2014). It has been found that most of class A β-lactamases are also regulated by LysR family regulators (see reviews of Bush et al., 1995; Philippon et al., 2016). Expressions of some inducible class A β-lactamase genes (e.g., *nmcA, cumA, smeA, cdiA, sed-1, hugA*, and *penA*) are ultimately controlled by cognate LysR family transcriptional regulators, and these β-lactamase genes located nearby *lysR* and transcribed divergently (Datz et al., 1994; Naas and Nordmann, 1994; Jones and Bennett, 1995; Naas et al., 1995; Trépanier et al., 1997; Petrella et al., 2001; Liassine et al., 2002; Poirel et al., 2009).

Previous studies have shown that the [Fe-S] cluster biosynthesis-related genes are involved in antibiotic susceptibility in *Escherichia coli*. Bactericidal agents (quinolones, aminoglycosides, and β-lactams) were proposed to induce oxidative stress and produce reactive oxygen species (ROS), thereby destabilizing the Fe–S clusters and resulting in Fe(II)-mediated Fenton reactions (Dwyer et al., 2009). Disruption of the Fe–S cluster biosynthesis genes *iscS, iscU, hscA, hscB*, and *fdx* increased susceptibility to various antibiotics (e.g., cephalosporins, penicillins, and glycopeptides) in *E. coli* (Liu et al., 2010). IscR was discovered as a negative regulator controlling the Fe–S biogenesis system (Schwartz et al., 2001). It is widely conserved in *Proteobacteria* and is proposed to be a member of the large Rrf2 family of winged helix-turn-helix (wHTH) transcription factors (Schwartz et al., 2001). Recently, we showed that a transposon (Tn5) insertion in a gene encoding Fe–S cluster assembly regulator (*iscR*) affected selenite susceptibility and antimonite oxidation in *Comamonas testosteroni* S44 (Zheng et al., 2014; Liu H.L. et al., 2015). IscR is also reported to regulate more than 40 genes that are involved in various cellular processes in *E. coli* (Giel et al., 2006, 2013; Haines et al., 2015). Thus, IscR may be associated with the regulation of antibiotic susceptibility.

*Comamonas testosteroni* strains are primarily environmental bacteria that play an important role in environmental decontamination, having the ability to transform heavy metals and degrade a variety of toxic aromatic pollutants (Liu L. et al., 2015). Recently, *C. testosteroni* strains have also been recognized as human pathogens with potential to cause blood, endocardial, and abdominal infections (Duran et al., 2015; Parolin et al., 2016). Empiric therapy includes use of intravenous antibacterials of β-lactams and fluoroquinolones, especially cefoxitin and ciprofloxacin (Duran et al., 2015; Parolin et al., 2016). *C. testosteroni* S44 was isolated from the soil of an antimony (Sb) mine and is resistant to multiple heavy metals (Xiong et al., 2011) and some antibiotics, including cefalexin, cefazolin, benzylpenicillin, and ampicillin (unpublished data). The objective of this study was to elucidate the IscR-/CzoR-mediated regulatory mechanism of a newly identified Class A β-lactamase CzoA in *C. testosteroni* S44. Based on a gene knock-out and its complementation, electrophoretic mobility shift assay (EMSA), DNase I footprinting, and *lacZ* reporter gene assays, we found that the LysR-type transcriptional regulator CzoR positively regulates *czoA* expression and that IscR enhances this regulatory effect through binding with the *czoR* promoter region.

## Materials and Methods

### Bacterial Strains, Plasmids, and Culture Conditions

Bacterial strains, plasmids, and oligonucleotide primers used in this study are shown in Supplementary Table S1. All strains were grown at 37°C in Luria-Bertani (LB, Oxoid, United Kingdom) broth unless otherwise stated. Mueller-Hinton (MH, Beijing Land Bridge Technology, China) broth dilution was used to determine the minimal inhibitory concentration (MIC) of antibiotics. Antibiotic disk (Hangzhou Microbial Reagent, China) diffusion tests were used for the antibiotic susceptibility assay (cephalexin and cefazolin). Appropriate antibiotic agents were added when preparing the seed liquid of all bacteria possessing a plasmid. Then, the seed liquid was used directly in relevant experiments.

### Bioinformatic Analysis

Whole-genome shotgun sequencing was performed using a Roche 454 Genome Sequencer FLX instrument as described previously (Xiong et al., 2011). Multiple amino acid sequence alignments of CzoA with representative Class A β-lactamases and CzoR with its homologs were conducted using Clustal Omega^1^. The IscR-binding site was analyzed by the online program MEME^2^ (Bailey and Elkan, 1994). The -35 and -10 sequences were predicted using Softberry BPROM Tool^3^ (Solovyev and Salamov, 2011).

### Construction of *iscR* and *czoR* Mutants and Complemented Strains

The *iscR*-mutant strain *iscR*-280 and its complemented strain *iscR*-280C were generated in our previous study (Liu H.L. et al., 2015). To create a *czoR-*mutant strain, the suicide allelic exchange vector pCM184-Cm was used as previously described (Chen et al., 2015). The upstream and downstream regions of *czoR* were amplified with the primer pairs M-*czoR*-up-F/M-*czoR*-up-R and M-*czoR*-down-F/M-*czoR*-down-R, respectively. Subsequently, the upstream and downstream PCR fragments were cloned into the *Aat*II–*Bsr*GI and *Apa*I–*Sac*I sites of pCM184-Cm, respectively. The resulting *czoR* allelic exchange vector pCM184-*czoR* was introduced into the strain S44 via biparental conjugation with the *E. coli* strain S17-1 (*pir*) (Simon et al., 1983), and the double crossover *czoR* mutant was selected using 50 μg/ml chloramphenicol and 25 μg/ml tetracycline. The tetracycline-sensitive and chloramphenicol-resistant mutant was then confirmed by PCR using primers *czoR*-inner-F/*czoR*-inner-R (Supplementary Table S1). For *czoR* complementation, the complete *czoR*-coding sequence was amplified via PCR and digested with *Xba*I and *Eco*RI. The fragment was subcloned into the broad host-range vector pCPP30, generating plasmid pCPP30::*czoR*. Then, the pCPP30::*czoR* plasmid was transferred into the Δ*czoR* strain by biparental conjugation (Simon et al., 1983) to yield the complemented strain Δ*czoR*-C.

### Purification of His_6_-CzoA, His_6_-IscR, and His_6_-CzoR

Expression and purification of the recombinant His_6_-CzoA, His_6_-IscR, and His_6_-CzoR proteins were conducted as described previously (Liu H.L. et al., 2015). Complete coding regions of CzoA, IscR, and CzoR were PCR amplified and subcloned into the His-tag expression vectors pET-28a(+) (CzoA and IscR) or pET-32a(+) (CzoR) (Novagen), yielding plasmids pET-28a(+)-CzoA, pET-28a(+)-IscR, and pET-32a(+)-CzoR (Supplementary Table S1). The recombinant plasmids were introduced via transformation into the *E. coli* strain BL21 (DE3). CzoA, IscR, and CzoR were overexpressed by adding 0.08 mM isopropyl β-D-1-thiogalactopyranoside (IPTG) at an OD_600_ of 0.2 and further culturing strains for 8 h at 28°C. Induced cells were then harvested by centrifugation and lysed in a French Press (JN-02C, JNBIO, China) in lysis buffer [50 mM Tris–HCl (pH 7.5) and 150 mM NaCl]. Soluble supernatant was mixed with 1 ml of nickel–nitrilotriacetic acid–agarose solution (Qiagen) and eluted in 1 ml of elution buffer [200 mM imidazole, 50 mM Tris–HCl (pH 7.5), and 150 mM NaCl]. After dialysis to remove imidazole, purified proteins were stored in 15% glycerol at -80°C. For *in vitro* enzymatic assay, His**_6_** tag of recombinant His_6_-CzoA were excised by addition of 0.5 U/ml bovine thrombin (Sigma–Aldrich, Buchs, Switzerland) and incubated at 4°C for 8 h, followed by dialysis in buffer [50 mM Tris–HCl (pH7.5) and 150 mM NaCl] (Guan et al., 2017).

### Enzyme Hydrolysis Assay and Inhibition of β-Lactamase Activity

Hydrolysis activities of 11 β-lactams (benzylpenicillin, ampicillin, cefalexin, cefazolin, cefuroxime, cefoxitin, ceftazidime, ceftriaxone, cefepime, meropenem, and imipenem) by CzoA without His_6_ tag were determined. His_6_-excised CzoA (0.01 μmol/l) and various concentrations (20–600 mmol/l) of each β-lactam were added to PBS buffer (pH 7.0), and incubated at 30°C for 30 min. The hydrolysis activities were evaluated through the changes in characteristic absorbance for the 11 β-lactams using a spectrophotometer (DU 800, Beckman, United States) (Lamoureaux et al., 2013). *K*_m_ values were determined by the Lineweaver–Burk plot (Bush and Sykes, 1986). Three technical and biological replicates were performed for each reaction.

Minimal inhibitory concentration profiles of the recombinant *E*. *coli* DH5α (Miller and Mekalanos, 1988) expressing *czoA* (pCT-Zori::*czoA*) and the isogenic strains of *C. testosteroni* S44 (S44, Δ*czoR*, Δ*czoR*-C, Δ*iscR*, and Δ*iscR*-C) were determined by the broth dilution method (Clinical and Laboratory Standards Institute, 2014) using 11 β-lactams. For the recombinant *E*. *coli* DH5α, two β-lactamase inhibitors, clavulanic acid, and tazobactam were also tested. *E. coli* DH5α (pCT-Zori) was used as a control. Clavulanic acid and tazobactam were generally fixed at a concentration of 2 and 4 μg/ml, respectively (Girlich et al., 2007; Naas et al., 2007; Lamoureaux et al., 2013; Clinical and Laboratory Standards Institute, 2014). Moreover, growth tendency of the isogenic strains of S44 under stress of 11 β-lactams with concentration below MIC was evaluated through OD_600_ values. Isogenic strains of *C. testosteroni* S44 in exponential growth phase were inoculated with each of 11 β-lactams in MH medium at 37°C and OD_600_ values were determined using a spectrophotometer after growth for 24 h.

### Disk Diffusion Susceptibility Testing

The disk diffusion method was used for an antibiotic susceptibility assay (Jorgensen et al., 1999). Antimicrobial disks impregnated with cefalexin or cefazolin were separately placed onto the inoculated MH agar plates. After being incubated at 37°C for 36 h, an inhibition zones around each antibiotic disk was measured. In addition, a spotting dilution assay was performed to determine the susceptibility of strains to cefalexin and cefazolin. Overnight cultures of strains S44, *iscR*-280, *iscR*-280C, Δ*czoR*, and Δ*czoR*-C were grown in LB medium. Tenfold gradient dilutions of these strains (OD_600_ = 1.0) were each plated (4 μl) onto solid LB medium containing 50 μg/ml of cefalexin or cefazolin. LB medium without antibiotics was used as a control. Agar plates were incubated at 37°C and photographed daily until colonies formed.

### Electrophoretic Mobility Shift Assay (EMSA)

The intergenic region of *czoR*–*czoA* was PCR amplified with primers EMSA-CzoR-F and EMSA-CzoR-R (Supplementary Table S1). The primer EMSA-CzoR-F was labeled with the fluorophore 5-carboxyfluorescein (FAM, Tsingke Biological Technology Company, Wuhan, China). To identify exact binding sequences of IscR, a 30 bp FAM-labeled DNA was synthesized (Tsingke Biological Technology Company, Wuhan, China) and directly annealed *in vitro*. For EMSA, the purified His_6_-CzoR and His_6_-IscR were each incubated with FAM-labeled DNA in 30 μl of incubation buffer [100 mM HEPES, pH 7.6, 5 mM ethylene diamine tetra acetic acid (EDTA), 50 mM (NH_4_)_2_SO_4_, 5 mM dithiothreitol (DTT), Tween 20, 1% (w/v), and 150 mM KCl] at 28°C for 30 min. After incubation, the mixtures were electrophoresed in an 8% native polyacrylamide gel in 1× Tris/Borate/EDTA (TBE) buffer for 1 h. The gels were then exposed using a phosphorimaging system (Fujifilm FLA-5100, United States).

### DNase I Footprinting Assay

To identify exact CzoR-binding sites within the intergenic region of *czoR–czoA*, a DNase I footprinting experiment was performed as described previously (Shi et al., 2017). The binding reaction was carried out in a 30 μl system containing 0 or 0.12 nM of purified CzoR and 100 ng of 5′-FAM labeled DNA fragment. After an incubation at 28°C for 30 min, DNase I (0.8 unit in 20 μl, Promega) was added to the binding mixture and incubated at 37°C for 10 min. Then, the reaction was terminated by adding 10 μl of 50 mM EDTA and an incubation in a water bath at 65°C for 10 min. Digested DNA fragments were purified using a NucleicSpin Gel and PCR Clean-up Kit (Macherey-Nagel, Germany) and analyzed with an Applied Biosystems 3730XL DNA Analyzer (Tsingke Biological Technology Company, Wuhan, China). Results were analyzed with GeneMarkerV1.6536 (Shi et al., 2017).

### *czoA*::*lacZ* Reporter Gene Assays

The *czoA* promoter (P*_czoA_*) region was amplified by PCR using the primers pLSP-czoA-F and pLSP-czoA-R (Supplementary Table S1). The PCR amplicon was then digested with *Eco*RI and *Bam*HI and directionally cloned into the *lacZ* reporter plasmid pLSPkt2lacZ, which was transformed into *E. coli* S17-1(*pir*) (Supplementary Table S1). The resulting plasmid pLSP-czoA was introduced into strains S44, *iscR*-280, *iscR*-280C, Δ*czoR*, and Δ*czo*R-C via biparental conjugation (Simon et al., 1983). All strains were inoculated into LB medium with or without addition of β-lactams. After being incubated at 37°C for 8 h, β-galactosidase activities were measured as previously described (Liu H.L. et al., 2015).

### Real-Time Quantitative RT-PCR

Each strain of S44, *iscR*-280, *iscR*-280C, Δ*czoR*, and Δ*czoR*-C was each inoculated into LB medium and incubated at 37°C for 8 h. Next, 0 or 25 μg/ml of cefalexin or cefazolin was added to the culture. After 1 h of induction, bacterial cells were harvested for total RNA extraction using TRIzol Reagent (Invitrogen, Grand Island, NY, United States) according to the manufacturer’s instructions (Invitrogen, Grand Island, NY, United States). Real-time RT-PCR was carried out using an Applied Biosystems^®^ ViiA^TM^ 7 Real-Time PCR System (Life Technologies, Carlsbad, CA, United States) and primers listed in Supplementary Table S1. Gene expression was normalized by the ΔΔCT method with an iQ5 Real-Time PCR Detection System (Bio-Rad, United States) (Pfaffl, 2001). An ATP-binding subunit encoding gene *clpX* (CTS44_RS19450) was used as a reference (Caille et al., 2014) and three technical and biological replicates were performed for each reaction. Statistically significant difference between control and treated samples was performed using Student’s *t*-test with *P* < 0.01 as borderline and *P* < 0.01 as statistically significant level. Data are expressed as the average of three experiments.

## Results

### Genetic Organization of *czoR* and *iscR*

In this study, a novel putative class A β-lactamase gene, we named *czoA* here (czo for cefazolin), was found from the draft genome of *C. testosteroni* S44 (ADVQ00000000.1, Xiong et al., 2011). CzoA consists of 301 deduced amino acids and shows high similarities with several established class A β-lactamases, PenA (AAB53622.1, 47%), NmcA (AOW71300.1, 43%), AST-1 (AAG44836.1, 43%), Sed-1 (WP_063864602.1, 43%), CdiA (CAA54738.1, 43%), BlaA (AIK22395.1, 43%), GIL-1 (WP_063860521.1, 41%), and HugA (AAL57765.1, 40%). A multiple sequences alignment showed that CzoA shares conserved residues (E^166^ and R^220^) and motifs (S^70^XXK^73^, S^130^DN, and K^234^TG) with other class A β-lactamases (Supplementary Figure S1). No obvious differences were detected among MICs of the wild-type strain S44 (Supplementary Table S2) and Δ*czoR* or Δ*iscR* (data not shown), possibly because there are two other putative β-lactamases of class B (WP_003070075.1) and class D (WP_034361410.1) in S44. Class B and Class D β-lactamases showed overlapping substrate profiles compared with Class A β-lactamase (Bush and Jacoby, 2010). Therefore, knock-out and complementation experiments of *czoA* were not performed.

**FIGURE 1:**
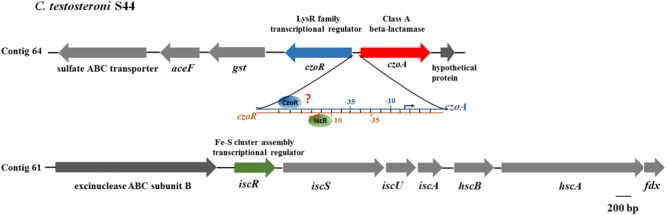
Genetic organization of *czoR* and *iscR.* The czo*R* and czo*A* genes form a divergent operon (*czoR*–*czoA*) located in contig 64. The *isc* operon of S44 is composed of the *iscRSUA*–*hscBA*–*fdx* genes and is located in contig 61. The diagram shows that both IscR and CzoR can bind to the *czoR–czoA* intergenic region, but the precise binding sites of CzoR are unknown. The –35 and –10 sequences were predicted using Softberry BPROM webtool (Solovyev and Salamov, 2011).

A LysR family transcriptional regulator encoding gene, here named *czoR*, located immediately upstream of *czoA* was also identified (**Figure 1**). CzoR displays the highest amino acid identity (59%) with AmpR of *Pseudomonas aeruginosa* using BlastP analysis (Caille et al., 2014). Sequence alignments of CzoR with AmpR (ADB64523.1, 59%), PenR (AAB53621.1, 57%), SedR (AAK63224.1, 56%), CdiR (CAA54736.1, 55%), HugR (AAL57764.1, 49%), and NmcR (AOW71475.1, 45%) were performed (Supplementary Figure S2). No putative CzoR-binding motifs were predicted using online program MEME, however, we found a putative CzoR-binding box in the promoter region of *czoA* using EMSA and DNase I footprinting assays, suggesting that CzoR may regulate *czoA* expression (**Figure 1**). In addition, to investigate the effect of the Fe–S cluster assembly regulator encoding gene *iscR* on antibiotic susceptibility, the Fe–S cluster biosynthesis-related genes were also analyzed. The S44 genome contains only one *isc* system, which is composed of the *iscRSUA*-*hscBA*-*fdx* genes located in contig 61 (**Figure 1**). IscR-binding motif varies among bacteria originated from different taxa (Novichkov et al., 2013). Based on IscR-binding motifs (5′-WTAMYYRNSNVDWWYRVWMRRBWWH-3′) in *C. testosteroni* KF-1 obtained from the RegPrecise database (Novichkov et al., 2013), we found a putative IscR-binding site within the *czoR*–*czoA* intergenic region using online program MEME (Bailey and Elkan, 1994), suggesting that IscR may also be involved in the regulation of *czoR*/*A*.

### Antibiotic Susceptibility

Considering *C. testosteroni* S44 contains multiple β-lactamases, we determined MICs of various β-lactam antibiotics against *E*. *coli* DH5α **(**pCT-Zori::*czoA***)** expressing CzoA. MIC tests using a conventional broth dilution method indicated that the recombinant strain produced the β-lactamase and its activity was inhibited by clavulanic acid or tazobactam. Determination of MICs of β-lactams for *E*. *coli* DH5α (pCT-Zori::*czoA*) showed that it was resistant to benzylpenicillin, ampicillin, and some cephalosporins such as cefalexin, cefazolin, cefuroxime, ceftriaxone, cefepime, and imipenem, but it remained susceptible to cefoxitin, ceftazidime, and meropenem (**Table 1**). The MICs of benzylpenicillin, ampicillin, ceftriaxone, and cefepime were significantly reduced by both tazobactam and clavulanic acid, while these of cefalexin, cefazolin, and cefuroxime were merely reduced by clavulanic acid (**Table 1**), and the others were not inhibited by tazobactam or clavulanic acid (data not shown in **Table 1**). It is interesting that MIC of ampicillin–tazobactam was 16-fold of that of ampicillin–clavulanic acid (**Table 1**), which indicated clavulanic acid is a sound inhibitor for class A β-lactamases, while tazobactam is a good inhibitor for other class of β-lactamase.

**Table 1:** β-Lactam activity against *E. coli* DH5α expressing CzoA.

	MIC (μg/ml)	
Antibiotic	*E. coli* DH5α (pCT-Zori::*czoA*)	*E. coli* DH5α (pCT-Zori)	Fold difference pCT-Zori::*czoA* /pCT-Zori
Benzylpenicillin	2048	8	256
Benzylpenicillin + TZB^a^	512	8	64
Benzylpenicillin + CLA^b^	64	8	8
Ampicillin	2048	2	1024
Ampicillin + TZB	128	2	64
Ampicillin + CLA	8	2	4
Cefalexin	1024	8	128
Cefalexin + CLA	128	8	16
Cefazolin	512	8	64
Cefazolin + CLA	32	8	4
Cefuroxime	128	8	16
Cefuroxime + CLA	8	8	1
Cefoxitin	8	8	1
Ceftazidime	2	2	1
Ceftriaxone	128	0.5	256
Ceftriaxone + TZB	8	0.5	16
Ceftriaxone + CLA	2	0.5	4
Cefepime	16	0.25	64
Cefepime + TZB	4	0.25	16
Cefepime + CLA	1	0.25	4
Meropenem	0.031	0.031	1
Imipenem	1	0.125	8
Tazobactam	32	32	1
Clavulanic acid	64	64	1

*^a^TZB, tazobactam at a fixed concentration of 4 μg/ml. ^b^CLA, clavulanic acid at a fixed concentration of 2 μg/ml; data are expressed as the average of three independent experiments.*

### CzoA Kinetics Analysis

Kinetic parameters of the CzoA β-lactamase obtained with the purified enzyme (without His_6_ tag) (Supplementary Figure S3) showed that CzoA had strong activities (*k*_cat_ values of 99–1085 s^-1^) to degrade benzylpenicillin, ampicillin, and cefazolin. Cefalexin, cefuroxime, ceftriaxone, and cefepime were hydrolyzed at low levels (*k*_cat_ values of 2–12 s^-1^), whereas hydrolysis of cefoxitin, ceftazidime, meropenem, and imipenem was not detectable (**Table 2**). These results are in agreement with antibiotic susceptibility testing that CzoA did not show obvious hydrolyzing activity for cefoxitin, ceftazidime, and meropenem. However, for imipenem, the MIC difference was observed (1 vs. 0.125 μg/ml), which indicates imipenem may be a substrate of CzoA (**Table 1**).

**Table 2:** Steady-state kinetic parameters for hydrolyses of β-lactam substrates by the native CzoA.

Substrate	*K*_m_ (mM)	*k*_cat_ (s^-1^)	*k*_cat_ (s^-1^)/*K*_m_ (mM^-1^ s^-1^)
Benzylpenicillin	2.17 ± 0.4	738 ± 14	349 ± 9
Ampicillin	5.69 ± 0.1	99 ± 3	18 ± 0.2
Cefalexin	0.33 ± 0.02	12 ± 1	36 ± 2
Cefazolin	2.25 ± 0.2	1085 ± 16	505 ± 20
Cefuroxime	0.37 ± 0.02	8 ± 0.5	22 ± 0.1
Cefoxitin	–	–	–
Ceftazidime	–	–	–
Ceftriaxone	0.24 ± 0.007	3 ± 0.05	13 ± 0.2
Cefepime	0.30 ± 0.02	2 ± 0.6	7 ± 0.9
Meropenem	–	–	–
Imipenem	–	–	–

*Data are expressed as the average of three independent experiments ± SD. –, not determinable.*

### Effects of *czoA/R* and IscR under Different Antibiotics

**FIGURE 2:**
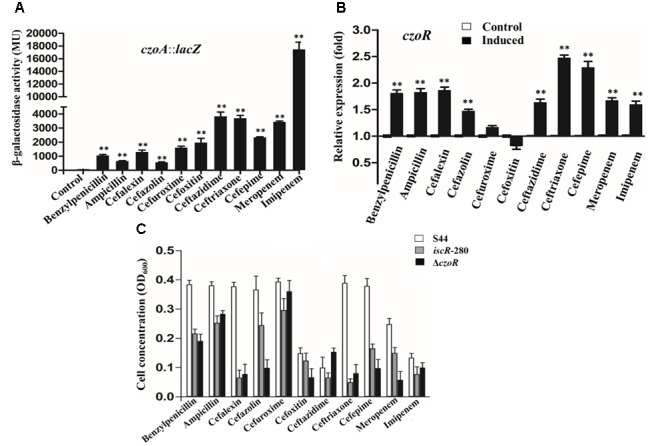
*czoA* and *czoR* were induced by different β-lactams. **(A)** A *lacZ* reporter fusion of *czoA*. Bacteria were cultured in LB medium with addition of 25 μg/ml benzylpenicillin, ampicillin, cephalexin, cefazolin, cefuroxime, cefoxitin, ceftazidime, ceftriaxone, cefepime, and 0.5 μg/ml meropenem and imipenem. A culture without β-lactams was used as a control. **(B)** Real-time RT-PCR results for *czoR* in S44. The relative *czoR* mRNA expression levels induced by 11 β-lactams (black bars) and in the control (without induction, white bars) were determined as described in the Section “Materials and Methods.” Error bars represent standard deviations of triplicate tests. The selected induction concentrations, which did not affect normal growth, were well below the MIC for the β-lactams. ^∗∗^Indicates a significant difference from the control (*p* < 0.01, Student’s *t*-test). **(C)** The growth tendency of the isogenic strains of S44 under the stress of the 11 β-lactams [benzylpenicillin (2048 μg/ml), ampicillin (2048 μg/ml), cephalexin (1024 μg/ml), cefazolin (2048 μg/ml), cefuroxime (128 μg/ml), cefoxitin (128 μg/ml), ceftazidime (128 μg/ml), ceftriaxone (1024 μg/ml), cefepime (512 μg/ml), meropenem (1 μg/ml), and imipenem (4 μg/ml)] was evaluated through the OD_600_ values.

Expression of *czoA::lacZ* was significantly induced by all 11 β-lactams tested (**Figure 2A**), although CzoA did not show obvious hydrolysis activity of cefoxitin, ceftazidime, meropenem, and imipenem *in vitro* (**Table 2**). Substrate induction experiments indicated that CzoA was an inducible class A β-lactamase in presence of β-lactams. Transcription level of *czoR* was significantly induced by nine β-lactams, but not inducible by cefuroxime and cefoxitin (**Figure 2B**); however, *czoR* still showed constitutive transcriptions without and with addition of cefuroxime and cefoxitin (**Figure 2B**).

To further investigate effects of CzoR and IscR on cephalosporin susceptibility, a mutant strain Δ*czoR* and its complemented strain Δ*czoR*-C were constructed. Successful deletion and complementation of *czoR* were confirmed by diagnostic PCR, as shown in Supplementary Figure S4. An *iscR*-mutant strain *iscR*-280 (Δ*iscR*) and a complemented strain *iscR*-280C (Δ*iscR-*C) were obtained from our previous study (Liu H.L. et al., 2015). Both mutant strains Δ*czoR* and Δ*iscR* showed significantly inhibited growth compared to S44 under a certain concentration of different β-lactams (**Figure 2C**). Antibiotic susceptibility phenotype of complemented strains Δ*czoR-*C and Δ*iscR-*C was mostly recovered (data not shown).

### Effects of *iscR* and *czoR* on Cefalexin and Cefazolin Susceptibility

**FIGURE 3:**
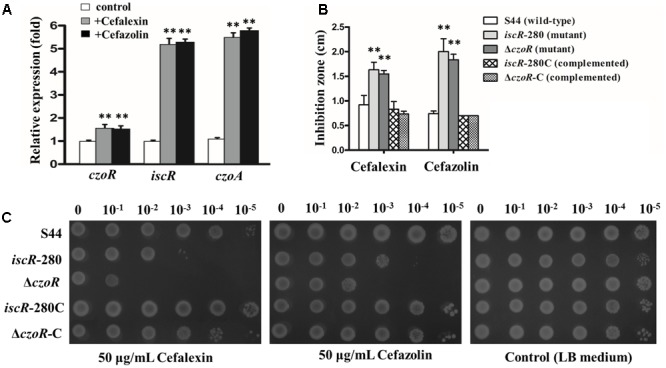
IscR and CzoR contribute to cephalosporin susceptibility. **(A)** Transcriptional levels of *czoR, iscR*, and *czoA* in S44 with or without (white bars) addition of 25 μg/ml of cefalexin (dark gray bars) and cefazolin (black bars). **(B)** Susceptibility tests of strains S44, *iscR-280, iscR*-280C, Δ*czoR*, and Δ*czoR-C* to different antibiotics (cefalexin and cefazolin) were determined using the Kirby–Bauer disk diffusion method. The inhibition zone of each disk was measured to the nearest millimeter. The diameter (0.7 cm) of susceptibility disks was counted in the inhibition zones. **(C)** Spotting assays for growths of strains S44, *iscR*-280, *iscR*-280C, Δ*czoR*, and Δ*czoR-*C on LB plates without antibiotics and those supplemented with 50 μg/ml cefalexin or cefazolin. Ten-fold serial dilutions of each culture were inoculated on the plates and incubated at 37°C for 48 h. Error bars represent standard deviations of triplicate tests. ^∗∗^Indicates a significant difference from the control (*p* < 0.01, Student’s *t*-test).

Real-time RT-PCR assays were performed in S44 with or without addition of cefalexin and cefazolin. Results showed that, similar to *czoA* and *czoR, iscR* expression was also significantly induced by cefalexin and cefazolin (**Figure 3A**), indicating *iscR* is also involved in cefalexin and cefazolin susceptibility. Kirby–Bauer disk diffusion assays showed that growth inhibition zones of Δ*czoR*- and *iscR*-280-mutant strains were significantly larger than S44 in presence of cefalexin and cefazolin (**Figure 3B**). Antibiotic susceptibility phenotypes of complemented strains were restored (**Figure 3B**). In addition, spotting assays showed that the *iscR*-280 strain, and to a greater extent for the Δ*czoR* strain, was more susceptible to cefalexin and cefazolin relative to the wild-type strain S44 (**Figure 3C**). Phenotypes of the complemented strains were restored, and all strains showed a similar growth trend on LB plates without antibiotics (**Figure 3C**). These results suggested that both IscR and CzoR are essential for cephalosporin susceptibility and that CzoR may play a more important role.

### CzoR Binds to the Promoter Region of *czoA*

**FIGURE 4:**
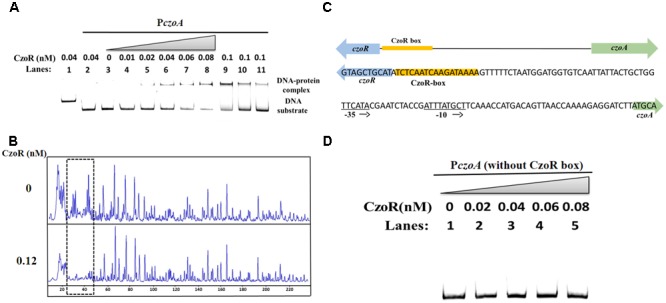
EMSA and DNase I footprinting analyses of CzoR interaction with P*czoA*. **(A)** Results of EMSA analysis of CzoR interaction with *PczoA*. Bands 1–3 indicate negative controls: 1, DNA probe containing no CzoR-binding motif (non-specific DNA probe) in the presence of CzoR; 2, DNA probe containing P*czoA* (specific DNA probe) in the presence of heat-inactivated CzoR; 3, specific DNA probe only; Bands 4–8 represent 0.01, 0.02, 0.04, 0.06, and 0.08 nM of CzoR incubated with a specific DNA probe; Bands 9–11 represent the competition assay using 1.8 pmol of the FAM-label promoter region of *czoA* and 0.1 nM CzoR competed against 0, 3.6, and 8.0 pmol of unlabeled promoter region of *czoA*. **(B)** Footprinting assay. The concentrations (nanomolar) of CzoR are indicated in the left lanes; reactions in each lane contained 100 ng of the FAM-labeled P*czoA* region with or without CzoR protein. The gridlines denote the regions protected by CzoR. **(C)** Sequence of the CzoR motif predicted by footprinting analysis. The CzoR box (yellow bars) and the P*czoA* sites (–35 and –10) are indicated. **(D)** EMSA for the DNA-binding activity of CzoR and P*czoA* without the CzoR box.

To examine interactions between CzoR and P*czoA*, EMSA was performed with a 259 bp fragment of P*czoA* and purified CzoR (Supplementary Figure S5). With an increasing CzoR concentration, free DNA substrates gradually disappeared, while intensity of shifted DNA bands increased (**Figure 4A**). Reactions using heat-denatured CzoR and a non-specific DNA probe did not show any lagging bands. Moreover, unlabeled *czoA* DNA substrates could competitively inhibit CzoR binding to the FAM-labeled *czoA* (**Figure 4A**). These results indicated that CzoR could specifically bind to the *czoA* regulatory region.

Subsequently, a DNase I footprinting assay was conducted to determine exact binding sites of CzoR. Results showed that the -88 to -72 region in P*czoA* was obviously protected from DNase I digestion (**Figure 4B**), indicating that a 17 bp fragment (5′-TCTCAATCAAGATAAAA-3′) upstream of *czoA* was a CzoR-binding box in S44 (**Figure 4C**). For further confirmation, interactions between CzoR and a 211 bp fragment of P*czoA* without CzoR-binding box were tested. EMSA results showed that there was no band shift without CzoR-binding box (**Figure 4D**). These experiments demonstrated that the LysR family regulator CzoR can regulate the class A β-lactamase gene *czoA* in S44.

### IscR Binds to the Regulatory Region of *czoR*–*czoA*

**FIGURE 5:**
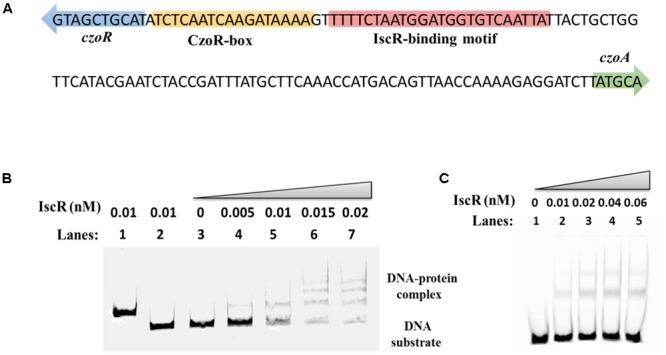
EMSA analysis for the IscR interaction with the *czoR*–*czoA* intergenic region. **(A)** IscR-binding motifs extracted from RegPrecise were used to identify potential IscR-regulated genes. A putative IscR-binding motif was located in the region between *czoR* and *czoA*. **(B)** EMSA analysis results for the IscR interaction with the *czoR*–*czoA* promoter. Bands 1–3 represent negative controls: 1, DNA probe containing no IscR-binding motif (non-specific DNA probe) in the presence of IscR; 2, DNA probe containing the putative IscR-binding motif (specific DNA probe) in the presence of heat-inactivated IscR; 3, specific DNA probe only; Bands 4–7 represent different concentrations of IscR added with a 1.8 pmol FAM-label of the DNA probe. **(C)** EMSA analysis results for the DNA-binding activity of IscR with a conserved 30 bp motif.

Based on IscR-binding motifs in *C. testosteroni* KF-1 obtained from the RegPrecise database (Novichkov et al., 2013), we found a putative IscR-binding site within *czoR*–*czoA* intergenic region. A putative IscR motif (TTTTCTAATGGATGGTGTCAATTAT) was located in the sense strand adjacent to the CzoR box (**Figure 5A**). The interaction between IscR and *czoR*–*czoA* intergenic region was examined by EMSA. With an increasing IscR concentration, lagging bands were clearly observed. In contrast, negative controls (non-specific DNA probe or heat-denatured IscR) did not show any lagging bands (**Figure 5B**). In addition, IscR was capable of binding to substrates containing a 30 bp sequence with a refined IscR-binding motif (**Figure 5C**). These data suggest that IscR can directly bind to the *czoR*–*czoA* promoter region, and may regulate the expression of both *czoR* and *czoA*.

### CzoR Is Essential for *czoA* Expression and IscR Positively Regulates *czoR* Expression

**FIGURE 6:**
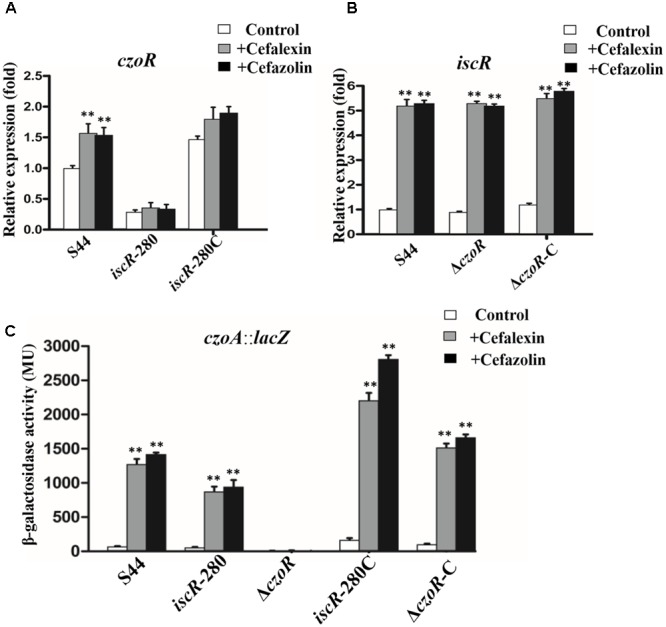
Transcriptional analysis. **(A,B)** Represents real-time RT-PCR results for *czoR* and *iscR*. The relative *czoR* and *iscR* mRNA expression levels induced by cefalexin (gray bars) and cefazolin (black bars) and in the control (without induction, white bars) were determined as described in the section “Materials and Methods.” Error bars represent standard deviations of triplicate tests. **(C)** Shows a *lacZ* reporter fusion of *czoA.* Bacteria were cultured in LB medium with or without (white bars) addition of 25 μg/ml cefalexin (gray bars) and cefazolin (black bars). ^∗∗^Indicates a significant difference from the control (*p* < 0.01, Student’s *t*-test).

To investigate how *iscR* and *czoR* influence each other and further affect *czoA* expression, real-time RT-PCR transcription analyses were performed using *C. testosteroni* S44 isogenic strains with or without addition of cefalexin or cefazolin. Deletion of *iscR* significantly decreased *czoR* expression, and *czoR* was not induced by cefalexin and cefazolin in *iscR*-280-mutant strain (**Figure 6A**). *czoR* deletion did not affect transcription level of *iscR* (**Figure 6B**) indicating that *czoR* induction by cefalexin and cefazolin was depend on IscR expression. Phenotypes of the complemented strain *iscR*-280C were recovered.

Subsequent efforts focused on *czoA* expression in the isogenic strains of *C. testosteroni* S44 using a *lacZ* reporter gene assay. Results showed that expression of *czoA::lacZ* was significantly induced by cefalexin and cefazolin in S44, consistent with induction expressions of *czoR* and *iscR* in presence of cefalexin and cefazolin (**Figure 3A**). Deletion of *iscR* (*iscR*-280) decreased expression of *czoA::lacZ* compared to S44 with addition of cefalexin and cefazolin (**Figure 6C**), possibly because transcription level of *czoR* was decreased in *iscR*-280 (**Figure 6A**). However, in *ΔczoR*, β-gal activity of CzoA::LacZ was almost non-existent, even at higher concentrations of cefalexin and cefazolin (50–100 μg/ml) or using different induction times (data not shown), indicating that *czoR* is essential for *czoA* expression. Although *iscR* expression was also induced by cefalexin and cefazolin in Δ*czoR*, IscR could not directly regulate *czoA* expression without CzoR. Higher β-gal activities in *iscR*-280C and Δ*czoR*-C compared to S44 may be attributable to a multicopy-based complementation used for both *iscR* and *czoR.* These results suggest that IscR may positively regulate *czoR* expression of and affect *czoA* expression, which provided a link between IscR and β-lactam susceptibility regulation.

## Discussion

CzoA, newly identified in *C. testosteroni* S44, hydrolyzed some penicillins and cephalosporins, and was inhibited by tazobactam or clavulanic acid (**Tables 1, 2**). Substrate and inhibition profiles are similar to those of several reported group 2b Class A β-lactamases (see review of Bush and Jacoby, 2010). Surprisingly, unlike most class A β-lactamases, kinetic parameters of purified CzoA β-lactamase showed that CzoA had hydrolysis activities against ceftriaxone and cefepime (**Table 2**). *k*_cat_ values of ceftriaxone (3 ± 0.05 s^-1^) and cefepime (2 ± 0.6 s^-1^) and low *K*_m_ values (0.24 ± 0.007 mM, 0.30 ± 0.02 mM) lead to relatively high *k*_cat_/*K*_m_ values (**Table 2**). Fold change of imipenem MICs between the strains *E. coli* DH5α (pCT-Zori::*czoA*) and *E. coli* DH5α (pCT-Zori) indicated CzoA showed resistance to imipenem, which is similar to carbapenem-hydrolyzing serine class A β-lactamases NmcA of *E. cloacae* NOR-1 and Sme-1 of *S. marcescens* S6 (Naas and Nordmann, 1994; Naas et al., 1995). However, CzoA did not confer resistance to meropenem in *E. coli*. Class B and Class D β-lactamases, existed in S44, may have overlapping functions compared with Class A β-lactamase, such as CzoA, since Class B and Class D β-lactamases showed hydrolysis activities to most β-lactams, including carbapenems, and cloxacillin/oxacillin/carbapenems, respectively (Bush and Jacoby, 2010).

In opposite orientation from the P*czoA*, a LysR-type transcriptional regulatory protein CzoR was identified (**Figure 1**). As is often the case for other LysR-regulated genes, genes encoding Class A β-lactamase and regulator were adjacent and opposite to one another, with overlapping and divergent promoters, which may provide tighter control of gene overexpression and prevent an inadvertent gene activation (Naas and Nordmann, 1994).

We observed that *czoA* expression was induced by 11 β-lactams and *czoR* was induced by 9 β-lactams and constitutively transcribed with or without cefuroxime and cefoxitin (**Figures 2A,B**). Such results are somehow in agreement with previous studies. For example, *cdiAR* operon encoding for Class A β-lactamase biosynthesis were also inducible by β-lactams in *E. coli* strains (Jones and Bennett, 1995). As for SmeR, β-lactams did not affect its expression (Naas et al., 1995). While in *C. gillenii*, no LysR-type regulatory gene was found upstream of the *bla*_GIL-1_ gene, which fits non-inducibility of β-lactamase expression (Naas et al., 2007). Although some β-lactams can induce AmpC, these β-lactams were not direct bind AmpR (Jones and Bennett, 1995).

Even though CzoR acting as a positive regulator for CzoA is similar to previous reported LysR-type regulators, such as CdiR, HugR, NmcR, PenR, SedR, and SmeR (Datz et al., 1994; Naas and Nordmann, 1994; Jones and Bennett, 1995; Naas et al., 1995; Petrella et al., 2001; Liassine et al., 2002; Poirel et al., 2009; Guérin et al., 2015), our results discovered a novel LysR-type (CzoR)-binding motif (5′-TCTCAATCAAGATAAAA-3′) in *C. testosteroni* S44 (**Figure 4**). Such motif is different from the reported AmpR-binding sites in *C. freundii, E. cloacae* NOR-1, *S. marcescens* S6, and *P. aeruginosa* (Lindquist et al., 1989; Naas and Nordmann, 1994; Naas et al., 1995; Balasubramanian et al., 2012).

In addition to the CzoR-binding motif, we also found a putative IscR-binding motif in the *czoR–czoA* intergenic region (**Figure 5**). Previous studies have shown that IscR is a global regulator involved in regulation of various physiological processes during growth and stress responses (Daung-nkern et al., 2010; Zheng et al., 2014; Liu H.L. et al., 2015), but little is known about the role of IscR in antibiotic susceptibility regulation interacting with β-lactamase. This study demonstrated that IscR indirectly influenced *czoA* expression through *czoR* regulation based on following observations (**Figures 2, 3, 5, 6**): (i) IscR/CzoR/CzoA was induced by cefalexin and cefazolin, and IscR could directly bind to the *czoR*–*czoA* promoter; (ii) *iscR* deletion decreased transcription level of *czoR* and *czoA*; (iii) *czoR* deletion had no effect on *iscR* transcription, although *czoA* expression was completely inhibited; and (iv) susceptibility to cefalexin and cefazolin was increased in Δ*iscR* and further increased in Δ*czoR*. CzoR, therefore, acted as a positive regulator for CzoA β-lactamase biosynthesis and IscR positive regulated *czoR* expression.

It has been shown that Fe–S cluster biosynthesis may also be involved in antibiotic susceptibility (Daung-nkern et al., 2010; Liu et al., 2010). Disruption of Fe–S cluster results in Fe(II)-mediated Fenton reactions and enhances oxidative stress. Our previous work showed that deletion of *iscR* significantly decreased cellular γ-glutamylcysteine ligase (γ-GCL) activity and glutathione (GSH) content (Liu H.L. et al., 2015), which play important roles in Fe–S cluster formation and H_2_O_2_ consumption, respectively (Qi et al., 2012; Wang et al., 2012). To guard against oxidative stress resulting from bactericidal agents, such as β-lactams, IscR may respond to β-lactam-induced stress, such as cefalexin and cefazolin, faster than CzoR.

In summary, our results reveal a novel mechanism in which CzoR positively regulates *czoA*, and IscR enhances the regulation by CzoR. Since IscR is a global regulator for cellular oxidative stress response, it is reasonable that IscR regulates expression of some β-lactamases, such as CzoA expression which is related to bacterial cell wall stress remission. Our study provides a new insight into the regulatory mechanism of class A β-lactamases and demonstrates, for the first time, that IscR is involved in antibiotic susceptibility via the regulation of *czoR*–*czoA*.

## Author Contributions

WZ and HL designed and performed the experiments and wrote the manuscript. JL wrote and revised the draft of the manuscript. LC participated in the experiments. GW designed the study and revised the draft of the manuscript. All authors read and approved the final manuscript.

## Conflict of Interest Statement

The authors declare that the research was conducted in the absence of any commercial or financial relationships that could be construed as a potential conflict of interest.

## Footnotes

1 http://www.ebi.ac.uk/Tools/msa/clustalo/

2 http://meme-suite.org/tools/meme

3 http://linux1.softberry.com/berry.phtml?topic=bprom&group=programs&subgroup=gfindb
