# Supplementary material for: Regulation of Class A β-Lactamase CzoA by CzoR and IscR in Comamonas testosteroni S44
Source: Front Microbiol. 2017 Dec 22;8:2573. doi: 10.3389/fmicb.2017.02573 (PMC5744064; doi:10.3389/fmicb.2017.02573)
Supplement: Supplementary file 1 [file Data_Sheet_1.DOCX]

***Supplementary Material***

**Regulation of class A *β*-lactamase CzoA by CzoR and IscR in** ***Comamonas testosteroni* S44**

Weiping Zhuang^a#^, Hongliang Liu^b#^, Jingxin Li^a^, Lu Chen^a^ and Gejiao Wang^a^*

State Key Laboratory of Agricultural Microbiology, College of Life Science and Technology, Huazhong Agricultural University, P.R. China^a^;

Shandong Provincial Research Center for Bioinformatic Engineering and Technique, School of life sciences, Shandong University of Technology, P.R. China^b^.

^#^ These authors contributed equally to this work.

* Correspondence:

*Prof. Gejiao Wang*

*State Key Laboratory of Agricultural Microbiology,*

*College of Life Science and Technology,*

*Huazhong Agricultural University Wuhan 430070, P. R. China*

Tel: +86-27-87281261; Fax: +86-27-87280670

E-mail: [gejiao@mail.hzau.edu.cn](mailto:gejiao@mail.hzau.edu.cn)

**Supplementary Figures and Tables**

**CzoA -----------MQRRSMLTTGLALGL-----GAWGLSGCAFISKQQAAAARTLSDELAAL 44**

**GIL-1 ------MPHFRL---ALIPLLTAFCL-----PAFA--------------HPTTLNKVKEA 32**

**NmcA -----MSLNVKQSRIAILFS---SCLISISF--FS------------QANTKGIDEIKNL 38**

**AST-1 MTFSALPFRRAD-RRRLLAAALAACALTLTAACDSGTVTVPV-TDSVTTSAVADPRFAEL 58**

**HugA ------MFKKTF-RQTAAIAVSLISLLASAT-LWAN------------TNNTIEAQLSEL 40**

**Sed-1 ------MLKERF-RQTVFIAAAVMPFIFSSTSLHAQA---------TSDVQQVQKKLAAL 44**

**CdiA ------MFKKRG-RQTVLIAA-VLAFFTASSPLLART---------QGEPTQVQQKLAAL 43**

**PenA ------MTHSSQ-RRILLLAAATAPLALSLGACAARD--ATV-SDAASPVGAAPASFAAL 50**

**BlaA ------MKHSSL-RRSLLLAGITLPLVSFALPAWA-----------NALPASVDKQLAEL 42**

**. .**

**CzoA EIQAQGRFGLYVLDTVSGAE-AGWRGDERFPMCSTFKTLLAARMLYLAQRDEIRLWRKLY 103**

**GIL-1 ESQLTARVGYAELDLTSGEILESYRLQERFPMMSTFKVLLCGAVLARVDAGKERLDRRIP 92**

**NmcA ETDFNGRIGVYALDTGSGKS-FSYRANERFPLCSSFKGFLAAAVLKGSQDNRLNLNQIVN 97**

**AST-1 ETTSGARLGVFAVDTGSGRT-VAHRADERFPMASTFKGLACGALLREHPLSTGYFDQVIH 117**

**HugA EKYNQGRLGVALINTEDNSQ-ITYRGEERFAMASTSKVMAVAAVLKESEKQAGLLDKNIA 99**

**Sed-1 EKQSGGRLGVALINTADNSQ-VLYRADERFAMCSTSKVMTAAAVLKQSETHDGILQQKMT 103**

**CdiA EKQSGGRLGVALINTADRSQ-ILYRGDERFAMCSTSKTMVAAAVLKQSETQHDILQQKMV 102**

**PenA ERAAGGRLGVCAIDTATGRR-ALHRADERFPFCSTFKAMLGAAVLAQSVAHPGLLQQRVT 109**

**BlaA ERNANGRLGVAMINTGNGTK-ILYRAAQRFPFCSTFKFMLAAAVLDQSQSQPNLLNKHIN 101**

*** .*.* :: * :** : *: * : . :* : : :**

**CzoA YSPSEVVAWSPISEKRAGANGGMTVQELCEAMVLVSDNTAANVLLE-ASGGPAALTQWLR 162**

**GIL-1 FSRRDLVEYSPVTEKHL--TDGMTVGELCDAAITMSDNTAANLLLT-AIGGPQGLTAFLR 149**

**NmcA YNTRSLEFHSPITTKYK--DNGMSLGDMAAAALQYSDNGATNIILERYIGGPEGMTKFMR 155**

**AST-1 YSAAELVEYSPVTETRV--ETGMTVRELCDAAITVSDNTAGNQLLK-LLGGPEGFTASLR 174**

**HugA IKKSDLVAYSPITEKHL--VTGMSLAQLSAATLQYSDNTAMNKILD-YLGGPSSVTQFAR 156**

**Sed-1 IKKADLTNWNPVTEKYV--GNTMTLAELSAATLQYSDNTAMNKLLA-HLGGPGNVTAFAR 160**

**CdiA IKKADLTNWNPVTEKYV--DKEMTLAELSAATLQYSDNTAMNKLLE-HLGGTSNVTAFAR 159**

**PenA YGRSDLVNYSPVTERHV--DTGMTVAELCAATIQYSDNTAANELMK-RIGGPAAVTAYAR 166**

**BlaA YHESDLLSYAPITRKNL--AHGMTVSELCAATIQYSDNTAANLLIK-ELGGLAAVNQFAR 158**

**.: *:: *:: ::. * : *** * * :: ** .. ***

**CzoA ELGDGITRLDRNEPSLNTALPGDERDTTTPQAMVQSLQKLLLGDVLEGYARALLQQWLVD 222**

**GIL-1 TTGDRVTRLDRWEPELNEALPGDKRDTTTPENMAQTLRQLLTGKILTTTSQQQLTHWMVT 209**

**NmcA SIGDEDFRLDRWELDLNTAIPGDERDTSTPAAVAKSLKTLALGNILSEHEKETYQTWLKG 215**

**AST-1 SLGDATSRLDRWETDLNTAIPGDERDTTTPAALAADYRALVVGDVLGAPERDQLKAWLVA 234**

**HugA SINDVTYRLDRKEPELNTAIHGDPRDTTSPIAMAKSLQALTLGDALGQSQRQQLVTWLKG 216**

**Sed-1 SIGDTTFRLDRKEPELNTAIPGDERDTTSPLAMAKSLRKLTLGDALAGPQRAQLVDWLKG 220**

**CdiA SIGDTTFRLDRKEPELNTAIPGDERDTTSPLAMAKSLHKLTLGDALAGAQRAQLVEWLKG 219**

**PenA SIGDDTFRLDRWETELNTALPGDLRDTTTPAAMAANLRVLVLGDALPPAQRAQLIEWLRG 226**

**BlaA SIGDQMFRLDRWEPDLNTARPNDPRDTTTPAAMAASMNKLVLGDALRPAQRSQLAVWLKG 218**

**.* **** * .** * .* ***::* :. . * *. * : *:**

**CzoA SRTGDKRVRAGMPGDWTVGGKTGS-GERGTACDTLIVWPTAQSAPLLVTAYLTGSPLDGA 281**

**GIL-1 DKVAGPLLRSVLPAGWFIADKTGA-GARGSRGIVAALGPDG-KPSRIVVIYITESQATMA 267**

**NmcA NTTGAARIRASVPSDWVVGDKTGSCGAYGTANDYAVVWPKN-RAPLIISVYTTKNEKEAK 274**

**AST-1 NTTGATRIRAGLPADWTVGDKTGS-PAYGSALDVAVAWPPG-RAPIVIAVLSTKSEQDAE 292**

**HugA NTTGDHSIKAGLPKHWIVGDKTGS-GDYGTTNDIAVIWPEN-HAPLILVVYFTQQEKDAK 274**

**Sed-1 NTTGGQSIRAGLPAHWVVGDKTGA-GDYGTTNDIAVIWPED-RAPLVLVTYFTQPQQDAK 278**

**CdiA NTTGGQSIRAGLPEGWVVGDKTGA-GDYGTTNDIAVIWPED-RAPLILVTYFTQPQQDAK 277**

**PenA NKVGDKRIRAGVPTGWRVGDKTGT-GDYGTTNDVGVLWPPS-RAPIVLAVYYTQTRADAK 284**

**BlaA NTTGDATIRAGAPTDWIVGDKTGS-GDYGTTNDIAVLWPTK-GAPIVLVVYFTQREKDAK 276**

**. .. ::: * * :..***: *: * :: ***

**CzoA GREAVLARAGEAIKRWYYTI---- 301**

**GIL-1 ERNRQIAGIGATLIQHWDE----- 286**

**NmcA HEDKVIAEASRIAIDNLK------ 292**

**AST-1 PDNALLAEATRVVVDALG------ 310**

**HugA YRKDIIAKATEIVTKEFANSPQTK 298**

**Sed-1 WRKDVLAAAAKIVTEGK------- 295**

**CdiA GRKDILAAAAKIVTEGL------- 294**

**PenA AKDDVIAAATRIASATLA------ 302**

**BlaA PRRDVLASVTKIILSQIS------ 294**

**:***

**Supplementary Figure S1.** Multiple amino acid sequence alignment of CzoA (WP_003067956.1) and representative class A *β*-lactamases*.* Nine class A *β*-lactamases alignments were conducted using Clustal Omega (<http://www.ebi.ac.uk/Tools/msa/clustalo/>), GenBank accession numbers and identities are given in parentheses: GIL-1 from *Citrobacter gillenii* (WP_063860521.1, 41%), NmcA from *Enterobacter cloacae* (AOW71300.1, 43%), AST-1 from *Nocardia asteroids* (AAG44836.1, 43%), HugA from *Proteus penneri* (AAL57765.1, 40%), Sed-1 from *Citrobacter sedlakii* (WP_063864602.1, 43%), CdiA from *Citrobacter diversus* (CAA54738.1, 43%). PenA from *Burkholderia cepacia* (AAB53622.1, 47%), BlaA from *Yersinia enterocolitica* (AIK22395.1, 43%). Letters highlighted in gray are conserved residues (E^166^ and R^220^) and motifs (S^70^XXK^73^, S^130^DN, and K^234^TG).

**NmcR ---MRARLPLNALRAFEASARYLNFTKAGLELHVSQAAVSQQVRTLEQMLGVALFTRVPR 57**

**AmpR --MVRRYLPLNPLRAFEAAARHLSFTRAAIELNVTHAAVSQQVRALEEQLGCVLFTRVSR 58**

**CzoR -----MQLPLNALRMFDAAARHQSLTRAAQELHVTQAAVSQHIRNLEERLGKPLFRRLPR 55**

**PenR MAKLRPHLPLNALRAFESSARHLNFTRAGLELSVTQAAVSQQVRALEERLGCALFTRLPR 60**

**HugR ---MRTHLPLNALRAFEASARHLNFTKAALELYVTQGAVSQQVRMLEDRLGVTLFKRLPR 57**

**SedR ---MRSHLPLNALRAFEASARHLSFTRAALELCVTQAAVSQQVRILEDRLNRVLFKRLPR 57**

**CdiR ---MRSNLPLNALRAFEASARHLSFTRAALELCVTQAAVSQQVRILEDRLNRVLFKRLPR 57**

****** ** *:::**: .:*:*. ** *::.****::* **: *. ** *: ***

**NmcR GLQLTDEGMHLLPSITEALQMMSSAMDKFHEGKIKEVLTIAVVGTFAIGWLLPRITAFLN 117**

**AmpR GLVLTHEGEGLLPVLNEAFDRIADTLECFSHGQFRERVKVGAVGTFAAGWLLPRLAGFYD 118**

**CzoR GLALTDEGQALWPVVAQSFERIEQSLQQVAEPRPREILTVGVVGTFAIGWLIPRLSQFQQ 115**

**PenR GLDLTDEGRALLPVLSDAFSRIETVLQQFDGGRLRQVLTLGVVGTFALGWLMPRLKRFRD 120**

**HugR GLEMTDDAQILFSVLTTAFSDIERVFKQFECGEYRDVVSIAAVGTFAVGWLLPRLAEFRQ 117**

**SedR GLEMTDEAQALFAVLTDAFGQIDTIFRQFEGGEYREVLTVAAVGTFAVGWLLPRIEQFRQ 117**

**CdiR GLEMTDEAQALFAVLTDAFGQIDTIFRQFEGGEYREVLTVAAVGTFAVGWLLPRIEQFRQ 117**

**** :*.:. * : :: : : . . :: :.:..***** ***:**: * :**

**NmcR ENPWIDIRILTHNNVVNLAAEGIDASIRFGTGGWINTENILLFQAPHTVLCSPETSKKLY 177**

**AmpR SHPHIDLHISTHNNHVDPAAEGHDYTIRFGNGAWHESDAELIFSAPHAPLCSPAIAEQLQ 178**

**CzoR LHPYIDLRLLTNNNRVDLAGEGLDAAVRFGDGAWHGTHAQMLLRAPLSPMCTPMLAQQLR 175**

**PenR THPFVELRLRTHNNVVDLAAEGLDFAIRFGQGNWPATRNERLFDAPLTALCAPEIARRLT 180**

**HugR LYPRIEVNLRTNNNVVNLATEGLDFAIRFGEGLWPLTHNKALFSAPLTVLCSPTTAKRLH 177**

**SedR AHPFVDLRLRTNNNVVNLAAEGLDFAIRFGSGQWPATHNEMLCEAPLTVLCSPDTAQRLS 177**

**CdiR AHPFVELRLRTNNNVVNLAAEGLDFAIRFGNGLWPATHNEMLFEAPLTVLCTPETAQRLR 177**

*** :::.: *:** *: * ** * ::*** * * : : ** : :*:* :.:***

**NmcR IPSDLKKVCLLRSYRKEEWNNWFKAAGIDPWTITG--PIFDSTRLMIDAVKLGDYAALVP 235**

**AmpR QPDDVHRFTLLRSFRRDEWSRWLDCAGGTPPSPSQPVMVFDTSLAMAEAAQLGAGVAIAP 238**

**CzoR EPADLARQTLLRSYRTQEWEGWFAGLDQAAPLARG--AMFDSSLTLAEAAAQGAGIALLP 233**

**PenR QPADLAHETLLRSYRTDEWLGWFDAAQLEPWTVNG--PVFDSSRLMVEAAMQGMGIALAP 238**

**HugR YPTDLINENLYRSYREDEWLKWFEKAEISPIKITG--SIFDSSRLMIESAIYEGGVALAP 235**

**SedR HPADLLQENLLRSYRADEWDAWFAAAGVSAERISG--AMFDSSRMMVETVIQSGGAALVP 235**

**CdiR RPADLLQENLLRSYRVDEWDNWFAAAGVTAERING--AVFDSSRLMVETVIHTGGAALVP 235**

*** *: . * **:* :** *: :**:: : ::. *: ***

**NmcR YHMFQKELNERSVAKPFEIYATLGGYWLTLQKSRVNHNSEALNVFKEWIIEHSREFVLKS 295**

**AmpR VCMFSRLLQSGALVQPFAAEITLGGYWLTRLQSRTE--TPAMQQFARWLLNTAAA----- 291**

**CzoR ARMFEHMLQQGRLVRPFAHEVDTGAYWLTYLKSRQA--SAALQTFRQWLMTQLQTD---- 287**

**PenR ACMFERELQLGLLARPLDIDVHAGGYWLTSLKSKSL--TPAMTLFRDWITAEASAAASAA 296**

**HugR AKMFSREIKNGQLVQPFKIEVEMGKYWLTYLKSKPM--TASMEIFQQWLINEALKECCE- 292**

**SedR AKMFSRELASGQLVRPFANEVDMGSYWLTHLKSKPV--TPAMEIFRDWIVKAE------- 286**

**CdiR AVMFARELAAGQLVRPFDIEIQMG-YWLTHLKSKPM--TPAMEIFRDWIVKMA------- 285**

**** : : :.:*: * **** :*: : :: * *:**

**Fig. S2.** Multiple amino acid sequence alignment of CzoR (WP_003067957.1) and it’s homologues. Seven LysR family proteins alignments were conducted using Clustal Omega (http://www.ebi.ac.uk/Tools/msa/clustalo/) (GenBank accession numbers and identity are given in parentheses): NmcR from *Enterobacter cloacae* (AOW71475.1, 45%), AmpR from *Pseudomonas aeruginosa* (ADB64523.1, 59%), PenR from *Burkholderia cepacia* (AAB53621.1, 57%), HugR from *Proteus penneri* (AAL57764.1, 49%), SedR from *Citrobacter sedlakii* (AAK63224.1, 56%), CdiR from *Citrobacter diversus* (CAA54736.1, 55%). Highlighted in gray are predicted helix-turn-helix motif (HTH).


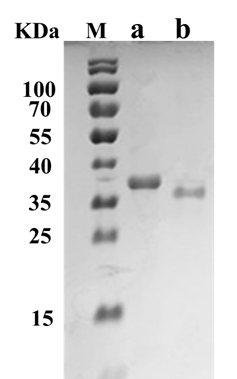


**Supplementary Figure S3.** The SDS-PAGE profile of purified proteins. Band a represents His_6_-CzoA; band b represent His_6_-excised CzoA. His tags were removed by bovine thrombin. M, represents protein marker.


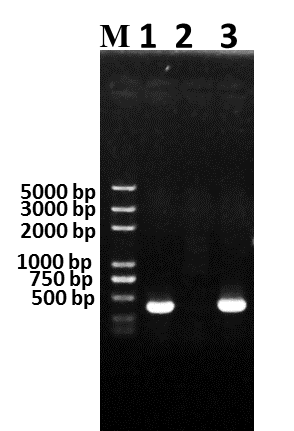


**Supplementary Figure S4.** Diagnostic PCR confirming the mutant strains S44-ΔczoR and complementation strain S44-ΔczoR-C. Lanes 1-3 represent PCR amplicons of strain S44, *czoR* mutant strain, and complementation strain S44-ΔczoR-C, respectively. M, represents the DNA ladder marker (DL 2000plus). Amplicon identities were confirmed by DNA sequencing.


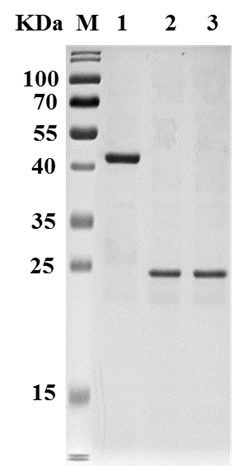


**Supplementary Figure S5.** The SDS-PAGE profile of purified proteins. Band 1 represents CzoR; band 2 represents IscR. M, represents protein marker.

**Supplementary Table S1 Strains, plasmids, and oligonucleotide primers used in this study**

| **Strains, plasmids,**  **or primers^a^** | **Genotypes, characteristics, or sequences (5**′**-3**′**)^b^** | **Source or references** |
| --- | --- | --- |
| **Strains** |  |  |
| ***Comamonas testosteroni***  S44 | Wild type, Rif^r^ | Xiong et al., 2011 |
| *iscR*-280 | *iscR* Tn5 insertion mutant, Rif^r^, Cm^r^ | Liu et al., 2015 |
| *iscR*-280C | *iscR* complementary strain, Rif^r^, Cm^r^, Tet^r^ | Liu et al., 2015 |
| Δ*czoR* | *czoR* replaced by Cm mutant, Rif^r^, Cm^r^ | This study |
| Δ*czoR*-C | Δ*czoR* complement with *czoR* genes, Rif^r^, Cm^r^, Tet^r^ | This study |
| ***Escherichia coli*** |  |  |
| S17-1 (λ*pir*) | *Tp^r^ Sm^r^ recA thi pro hsdR^-^ hsdM^+^* RP4, 2Tc, Mu, Km, T7, λ*pir* | Simon et al., 1983 |
| DH5α (λ*pir*) | Φ80d*lacZ* ΔM15 Δ(*argF-lacZYA*) *U169 relA1 hsdR17 deoR thi-1 supE44 gyrA96 recA1/*λ*pir* | Miller and Mekalanos, 1988 |
| DH5α (pCT-Zori::*czoA*) | DH5α containing pCT-Zori with CzoA coding region, Cm^r^ | This study |
| DH5α (pCT-Zori) | DH5α containing pCT-Zori, Cm^r^ | This study |
| BL21(DE3) | F^-^ *omp*T *hsd*S_B_ (r_B_^-^m_B_^-^) *gal dcm* (DE3) | Liu et al., 2015 |
| BL21-CzoA | CzoA expression strain, Km^r^ | This study |
| BL21-IscR | IscR expression strain, Km^r^ | Liu et al., 2015 |
| BL21-CzoR | CzoR expression strain, Amp^r^ | This study |
| **Plasmids** |  |  |
| pCT-Zori | Broad host range, Cm^r^ | Chen et al., 2015 |
| pCT-Zori::*czoA* | pCT-Zori with CzoA coding region, Cm^r^, | This study |
| pCPP30 | Broad host range, Tet^r^ | Liu et al., 2015 |
| pCPP30::*czoR* | pCPP30 with CzoR coding region, Tet^r^ | This study |
| pLSP-kt2lacZ | LacZ-fusion vector, oriV, Km^r^ | Liu et al., 2015 |
| pLSP-*czoA* | pLSP-kt2lacZ containing *czoA* promoter | This study |
| pET-28a(+)  pET-28a(+)-CzoA | His_6_ Tag expression vector, Km^r^  pET-28a(+) with CzoA coding region, Km^r^ | Novagen  This study |
| pET-28a(+)-IscR | pET-28a(+) with IscR coding region, Km^r^ | Liu et al., 2015 |
| pET32a(+) | His_6_ Tag expression vector, Amp^r^ | This study |
| pET32a(+)-CzoR | pET-32a(+) with CzoR coding region, Amp^r^ | This study |
| pCM184-Cm | Km of pCM184 was replaced by Cm | Chen et al., 2015 |
| pCM184-*czoR* | pCM184 containing *czoR* upstream and  downstream fragments | This study |
| **Primers^a^** |  |  |
| EMSA-CzoR-F | CAAGGCGTTCAGTGGTAG | This study |
| EMSA-CzoR-R | ACTCGTCGCTCAATGTTCT | This study |
| EMSA-dCzoR-F | TCTAATGGATGGTGTCA | This study |
| RT-clpX-F | CACCAATATCCTGTTCAT | This study |
| RT-clpX-R | AATGATGCCGAACTTGAT | This study |
| RT-iscR-F | CAAGGAAAACTGTCTGGGCG | Liu et al., 2015 |
| RT-iscR-R | TGCGTTCACACGGATGGG | Liu et al., 2015 |
| RT-czoR-F | CGAAATTCTGACCGTGGG | This study |
| RT-czoR-R | AGCAGCCGAAGATCTATA | This study |
| RT-czoA-F | GAGAACATTGAGCGACGAG | This study |
| RT-czoA-R | AGAGCTTGCGCCACAGA | This study |
| *czoR*-inner-F | AGAGCTTTGAGCGCATAGAG | This study |
| *czoR*-inner-R | CAAACCAGCCTTCCCATT | This study |
| pLSP-czoA-F | AAAGAATTCGCGATTGCTCTATGCG | This study |
| pLSP-czoA-R | AAAGGATCCTGCTGCGGCTTGCT | This study |
| IscR-pro-F | AAAGGATCCATGCGTCTTACGACCAAAGG | Liu et al., 2015 |
| IscR-pro-R | AAAAAGCTTTATCGGAGTCGGGCTTCA | Liu et al., 2015 |
| pCT-czoA-F | AAA AAGCTTGGCAAGGCGTTCAGTGG | This study |
| pCT-czoA-R | AAA GGATCCTGGCAGCAAACCTCAAGC | This study |
| pET28A-CzoA-F | AAA GGATCCATGCAGCGACGCAGCATG | This study |
| pET28A-CzoA-R | AAA AAGCTTTGGCAGCAAACCTCAAGC | This study |
| pET32A-CzoR-F | AAAGAATTCATGCAGCTACCACTGAACG | This study |
| pET32A-CzoR-R | AAAAAGCTTCCTGTGCGGCTTAGATG | This study |
| Pcpp30-CzoR-F | AAATCTAGAATGCAGCTACCACTGAACG | This study |
| Pcpp30-CzoR-R | AAAGAATTCCCTGTGCGGCTTAGATG | This study |
| M-*czoR*-up-F | AAAGACGTCCAACGCCGCCAACT | This study |
| M-*czoR*-up-R | AAATGTACATCTTCGAGATTACGGATGT | This study |
| M-*czoR*-down-F | AAAGGGCCCGCCTACTGGCTGACCTATC | This study |
| M-*czoR*-down-R | AAAGAGCTCAGTGCCTGTGGCTTGTT | This study |

^a^ The primers were designed using the genomic sequence of the *C. testosteroni* S44 genome with the accession number of ADVQ00000000 (Xiong et al., 2011).

^b^ Restriction sites are underlined. Rif^r^, rifampicin resistant; Cm^r^, chloramphenicol resistant; Tet^r^, tetracycline resistant; Km^r^, kanamycin resistant; Amp^r^, ampicillin resistant.

**Supplementary Table S2 MIC profile of *C. testosteroni* S44**

| Antibiotic | MIC (μg/ml) |
| --- | --- |
| Benzylpenicillin | >2048 |
| Ampicillin | >2048 |
| Cefalexin | >1024 |
| Cefazolin | >2048 |
| Cefuroxime | >128 |
| Cefoxitin | 128 |
| Ceftazidime | 128 |
| Ceftriaxone | >1024 |
| Cefepime | >512 |
| Meropenem | 1 |
| Imipenem | 8 |
| Vancomysin | >2048 |
